# Photodynamic Inactivation of Human Coronaviruses

**DOI:** 10.3390/v14010110

**Published:** 2022-01-08

**Authors:** Brett A. Duguay, Adrian Herod, Eric S. Pringle, Susan M. A. Monro, Marc Hetu, Colin G. Cameron, Sherri A. McFarland, Craig McCormick

**Affiliations:** 1 Department of Microbiology & Immunology, Dalhousie University, 5850 College Street, Halifax, NS B3H 4R2, Canada; bduguay@dal.ca (B.A.D.); adrian.herod@dal.ca (A.H.); eric.pringle@dal.ca (E.S.P.); 2 Photodynamic, Inc., 1344 Summer Street, Halifax, NS B3H 0A8, Canada; susan@photo-dynamic.com (S.M.A.M.); marc@photo-dynamic.com (M.H.); colin.cameron@uta.edu (C.G.C.); 3 Department of Chemistry and Biochemistry, The University of Texas at Arlington, Arlington, TX 76019-0065, USA

**Keywords:** antiviral, coronavirus, emodin, HCoV-229E, HCoV-OC43, lentivirus, natural product, PhytoQuin, photodynamic inactivation, photosensitizer, ROS, SARS-CoV-2, spike

## Abstract

Photodynamic inactivation (PDI) employs a photosensitizer, light, and oxygen to create a local burst of reactive oxygen species (ROS) that can inactivate microorganisms. The botanical extract PhytoQuin^TM^ is a powerful photosensitizer with antimicrobial properties. We previously demonstrated that photoactivated PhytoQuin also has antiviral properties against herpes simplex viruses and adenoviruses in a dose-dependent manner across a broad range of sub-cytotoxic concentrations. Here, we report that human coronaviruses (HCoVs) are also susceptible to photodynamic inactivation. Photoactivated-PhytoQuin inhibited the replication of the alphacoronavirus HCoV-229E and the betacoronavirus HCoV-OC43 in cultured cells across a range of sub-cytotoxic doses. This antiviral effect was light-dependent, as we observed minimal antiviral effect of PhytoQuin in the absence of photoactivation. Using RNase protection assays, we observed that PDI disrupted HCoV particle integrity allowing for the digestion of viral RNA by exogenous ribonucleases. Using lentiviruses pseudotyped with the SARS-CoV-2 Spike (S) protein, we once again observed a strong, light-dependent antiviral effect of PhytoQuin, which prevented S-mediated entry into human cells. We also observed that PhytoQuin PDI altered S protein electrophoretic mobility. The PhytoQuin constituent emodin displayed equivalent light-dependent antiviral activity to PhytoQuin in matched-dose experiments, indicating that it plays a central role in PhytoQuin PDI against CoVs. Together, these findings demonstrate that HCoV lipid envelopes and proteins are damaged by PhytoQuin PDI and expands the list of susceptible viruses.

## 1. Introduction

Coronaviruses (CoVs) are enveloped viruses with large positive-sense, single-stranded RNA genomes. Human CoVs occupy two genera, the alphacoronaviruses (HCoV-NL63 and HCoV-229E) and betacoronaviruses (HCoV-OC43, HCoV-HKU1, Middle East respiratory syndrome–related coronavirus (MERS-CoV), severe acute respiratory syndrome-related coronavirus (SARS-CoV and SARS-CoV-2). CoV Spike (S) proteins enable attachment to diverse cell surface receptors and entry via receptor-mediated endocytosis [1]. Following uncoating in the cytoplasm, the CoV (+)ssRNA genome is translated into long polyproteins that are processed by viral proteases to yield non-structural proteins, including the viral RNA-dependent RNA polymerase (RdRp) and associated RNA modifying enzymes [2], as well as an RNA exonuclease enzyme that provides essential proofreading activity to ensure high-fidelity replication of the viral genome [3]. These viral enzymes assemble to form the replicase-transcriptase complex that orchestrates viral transcription and genome replication in discrete cytoplasmic compartments [4]. The RdRp uses the template (+)ssRNA genome to create full-length (−)ssRNA intermediates that can be copied into full-length genomic (+)ssRNA, as well as subgenomic (−)ssRNAs that can be copied into mRNAs that encode structural proteins: S, membrane, envelope, and nucleocapsid (N), as well as lineage-specific accessory proteins that aid immune evasion [5]. Newly synthesized viral genomes are coated with N proteins and assemble with the remaining structural proteins by budding into the ER-Golgi Intermediate Compartment (ERGIC). According to the longstanding model, progeny CoV virions escape the host cell by traversing the secretory pathway, but recent studies of SARS-CoV-2 and the related betacoronavirus mouse hepatitis virus have provided evidence for an alternative lysosomal egress pathway [6].

The emergence of SARS-CoV and MERS-CoV from animal reservoirs, followed by the current SARS-CoV-2 pandemic, has spurred efforts to develop safe and effective antivirals for coronaviruses. Moreover, there is a growing recognition that pandemic preparedness requires the identification and development of broadly acting antivirals that can target outbreak-prone families of viruses [7]. Broadly acting antivirals must target highly conserved viral enzymes or structural components of these families of viruses and drug candidates must be tested against representative strains across the phylogenetic tree.

One of the earliest examples of a broadly acting antiviral mechanism was photodynamic inactivation (PDI), a term first used 90 years ago by Clifton to describe the antiviral effects of methylene blue dye against bacteriophage [8]. Clifton borrowed the term “photodynamic” after observing that methylene blue only displayed antiviral activity in the presence of oxygen and light. Perdrau and Todd confirmed Clifton’s findings and extended them to include PDI of herpes simplex virus and vaccinia virus [9,10]. Since these seminal discoveries, many additional photosensitizing molecules have been identified for use in PDI of microbes, including many natural products [11]. PDI mechanisms are now more fully understood, whereby exposure of a photosensitizer (PS) to visible light and oxygen results in the generation of singlet oxygen and other reactive oxygen species (ROS) [12,13,14]. The photosensitization process, coupling PS activation and subsequent oxidative reactions, occurs via two routes: type I and type II reactions. In type I reactions, the excited PS participates in electron transfer reactions involving oxygen, which result in the formation of superoxide and hydroxyl radicals. Conversely, type II reactions result in the formation of singlet oxygen from energy transfer between the excited PS and molecular oxygen. Ultimately, the ROS produced will act over short distances to damage proteins through irreversible modifications such as carbonylation, cause nucleic acid oxidation leading to strand damage or mutations, or negatively affect lipid membrane fluidity and integrity following lipid peroxidation [15,16]. Such damage to macromolecules over short distances is particularly effective in inactivating viruses, which, unlike prokaryotes and eukaryotes, are unable to repair damage once it occurs.

The plant extract PhytoQuin^TM^ (previously referred to as Orthoquin) is a powerful PS with antimicrobial properties [17,18,19]. We previously demonstrated that PhytoQuin inactivates enveloped herpes simplex virus type 1 (HSV-1) and HSV-2 and non-enveloped human adenovirus type 5 (hAd5), in a dose- and light-dependent manner [20]. Because photoactivated PhytoQuin inactivated both enveloped and non-enveloped viruses, we reasoned that it likely disrupts the function of surface-exposed viral attachment proteins [20]. Considering its proposed mechanism of action in damaging macromolecules at short distances, we reasoned that PhytoQuin PDI could also inactivate emerging viruses of concern like CoVs. Here, using the common cold CoVs, HCoV-OC43 and HCoV-229E, as well as pseudotyped lentiviruses bearing SARS-CoV-2 S protein, we demonstrate the antiviral effect of PhytoQuin PDI against CoVs. We provide evidence for antiviral mechanism of action by measuring damage to CoV S proteins, lipid envelopes and RNA. We also demonstrate that the PhytoQuin constituent emodin is sufficient to act as a PS and inactivate coronaviruses.

## 2. Materials and Methods

### 2.1. Cells, Viruses, and Reagents

Human embryonic kidney (HEK) 293T and 293A cells, and Huh7.5 (human hepatoma) cells were cultured in Dulbecco’s modified Eagle’s medium (DMEM; 11965118, Thermo Fisher Scientific, Grand Island, NY, USA) supplemented with 10% fetal bovine serum (FBS; A31607-02, Thermo Fisher Scientific), 100 U/mL penicillin/100 µg/mL streptomycin (Pen/Strep; 15140-122, Thermo Fisher Scientific), and 20 mM L-glutamine (Gln; 25030-081, Thermo Fisher Scientific). HCT-8 (human adenocarcinoma) cells were cultured in 10% FBS/DMEM + Pen/Strep/Gln and further supplemented with 1X MEM Non-Essential Amino Acids (NEAA; 11140050, Thermo Fisher Scientific). BHK-21 (baby hamster kidney) and Vero (African green monkey kidney) cells were cultured in 5% FBS/DMEM + Pen/Strep/Gln. HEK 293A cells stably expressing angiotensin-converting enzyme 2 (ACE2) and transmembrane serine protease 2 (TMPRSS2) were generated by sequential transduction with pLJM1-based ACE2 and TMPRSS2 lentiviral vectors with selection using puromycin (Puro) and blasticidin (BSD), respectively. All cells were maintained at 37 °C in a 5% CO_2_ atmosphere.

pLJM1-ACE2-BSD was cloned from Caco-2 cell cDNA using primers: ACE-F 5′-AATTACCGGTATGTCAAGCTCTTCCTGGC and ACE2-R 5′-AATTGTCGACCTAAAAGGAGGTCTGAACATCATCAG. pLJM1-TMPRSS2-V5-Puro was generated by subcloning from pLVX_317 (Broad Institute GPP TRCN0000467270) into pLJM1 [21] using NheI and MluI restriction endonucleases. Lentiviruses were generated by transfecting HEK 293T cells with lentivirus backbone pLJM1, a Gag-Pol encoding packaging vector (psPAX2, a kind gift form Didier Trono; Addgene #12260), and plasmid encoding VSV glycoprotein (pMD2.G, a kind gift from Didier Trono; Addgene #12259) using polyethyleneimine MAX^®^ (Polysciences, Warrington, PA, USA). Lentiviruses were harvested 48 h after transfection, filtered with a 0.45 µm filter, and stored at −80 °C until use.

Stocks of human coronavirus OC43 (HCoV-OC43; VR-1558, ATCC) were propagated in Vero cells. Cells were infected at a MOI of 0.05 for 1 h at 33 °C in serum-free DMEM. After 1 h, the infected cells were maintained in DMEM supplemented with 1% FBS and Pen/Strep/Gln for five days at 33 °C. Upon harvest, the culture supernatant was centrifuged at 1000× *g* for 5 min at 4 °C, the supernatant aliquoted, and then stored at −80 °C. Stocks of human coronavirus 229E (HCoV-229E; VR-740, ATCC) were prepared using a similar protocol, using Huh7.5 cells and DMEM supplemented with 2.5% FBS and Pen/Strep/Gln.

The preparation of PhytoQuin™ from an ethanolic extract of *Polygonum cuspidatum* has been previously described [20]. PhytoQuin (provided by PhotoDynamic Inc., Lot# SMKW115, Halifax, NS, Canada) and emodin (13109, Cayman Chemical, Ann Arbor, MI, USA) were solubilized in DMSO. All oligonucleotides were synthesized by Thermo Fisher Scientific (Pleasanton, CA, USA).

### 2.2. AlamarBlue Cytotoxicity Assay

BHK-21 cells and Huh7.5 cells were seeded in 96-well plates at 1 × 10^4^ cells/well in 5% FBS/DMEM + Pen/Strep/Gln or 10% FBS/DMEM + Pen/Strep/Gln, respectively. The following day, the media were removed and 2-fold dilutions of PhytoQuin (from 2.4 to 78 μg/mL for BHK-21; 0.125 to 64 μg/mL for Huh7.5) or equivalent DMSO concentrations (from 0.024 to 0.78% for BHK-21; 0.016 to 8% for Huh7.5) diluted in medium (1% FBS/DMEM + Pen/Strep/Gln, BHK-21; 2.5% FBS/DMEM + Pen/Strep/Gln, Huh7.5) were added to the cell monolayers and incubated 20 cm beneath a 30 W light-emitting diode (LED) visible light lamp (HC-GTG21-30W, SOLLA) for up to 30 min at room temperature. “Dark” and “Light” treatments were performed with 96-well plates wrapped or not wrapped in aluminum foil, respectively. The total fluence delivered during the 15 min or 30 min light treatments were 49.2 J/cm^2^ or 98.5 J/cm^2^, respectively, at a rate of 54.7 mW/cm^2^. After the incubation beneath the lamp, the cells were left for 44 h. At 44 h post-treatment, the media were replaced with fresh low-serum media containing 10% alamarBlue and placed back in the incubator for 3.5 h. Fluorescence was recorded using a FLUOstar Omega microplate reader (BMG Labtech, Ortenberg, BW, DE; ex/em: 544/590 nm) and normalized to fluorescence in the live cell control. The phototoxic concentration (CC_50_) for each cell line was calculated using GraphPad Prism 9.2.0 with a non-linear fit of [inhibitor] vs. response (variable slope, four parameters).

### 2.3. Photodynamic Inactivation of Viral Inocula

HCoV and lentiviral inocula diluted in serum-free DMEM supplemented with PhytoQuin, emodin, or DMSO were incubated 20 cm beneath a 30 W LED lamp in LiteSafe^®^ black 1.5 mL microtubes (06333-80, Cole-Parmer; Montreal, QC, Canada) for “Dark” treatments or in clear 1.5 mL microtubes (82050-504, VWR, Mississauga, ON, Canada) microcentrifuge tubes for “Light” treatments for the indicated times. After light or dark treatments, the viral inocula were used as indicated in Section 2.4, Section 2.5, Section 2.6, Section 2.7 and Section 2.8

### 2.4. HCoV-229E and HCoV-OC43 TCID50 Assays

BHK-21 cells and Huh7.5 cells were seeded in 96-well plates at a density of 1 × 10^4^ cells/well, respectively. The following day, the media were removed, and the cells were inoculated with 50 µL of serially diluted PhytoQuin-, emodin-, or DMSO-treated HCoV (OC43 on BHK-21 cells; 229E on Huh7.5 cells) as indicated in Section 2.3 and incubated for 1 h with shaking every 15 min. After infection, inocula were removed and replaced with low-serum media (see Section 2.2), and cells were incubated for 5 days to allow for the accumulation of cytopathic effect (CPE). To visualize CPE for TCID_50_ calculations, the media were removed, and the cells were washed once with phosphate-buffered saline (PBS) and agitated to dislodge infected cells. The cells were then fixed with methanol for 15 min and stained with 0.5% crystal violet (C0775, Sigma-Aldrich, Oakville, ON, Canada; diluted in a 1:1 solution of methanol and water) for 1 h. After staining, the crystal violet was rinsed off with water, and the plates were dried. The median tissue culture infectious dose (TCID_50_) was determined using the Spearman–Kärber method [22].

### 2.5. Transmission Electron Microscopy

Cell supernatants containing HCoV-OC43 particles were concentrated by centrifugation at 39,000 rpm in an SW-41 rotor with an Optima™ L-90K Ultracentrifuge (Beckman Coulter) for 2 h. Pelleted virus was resuspended in 100 µL of PBS. A volume of 80 uL of the preparation was diluted to 400 uL with serum-free DMEM, divided equally into clear or black microtubes and treated with Phytoquin or DMSO vehicle control as described in Section 2.3 above. Small drops (~30 µL) were placed onto Formvar/Carbon coated grids and left to settle for 10 min before rinsing the grid with distilled water. The grids were quickly rinsed with a small drop (~30 µL) of 2% uranyl acetate then stained for 30 s in a second drop. The stain was then wicked off on filter paper and left to dry completely. Grids were imaged with a JEM 1230 transmission electron microscope (JEOL) running at 80 kV and an ORCA-HR camera (Hamamatsu). Sample preparation, fixation, and TEM imaging were performed twice with representative images from one independent experiment shown.

### 2.6. RNase-Protection Assays and qPCR

HCoV-229E without treatment or HCoV-229E treated with 4 µg/mL PhytoQuin or 0.5% DMSO and LED light treatment at 49 J/cm^2^ as described in Section 2.3 were used for RNA extractions. Each sample contained 25 µL of virus, 1 ng in vitro transcribed *GFP* RNA (prepared from a T7-driven *GFP* plasmid using the HiScribe™ T7 High Yield RNA Synthesis Kit (E2040S, New England BioLabs)), and either serum-free DMEM only (“−“), serum-free DMEM with 500 µg RNase A/T1 (EN0551, Thermo Fisher Scientific) (“+R”), or serum-free DMEM with 500 µg RNase A/T1 and 0.1% Triton X-100 (“+T/R”). All samples were incubated at 37 °C for 20 min, after which TRIzol^®^ reagent (15596026, Thermo Fisher Scientific) was immediately added to arrest RNase activity. Then, 10 µg of 293T cell RNA (used as a carrier) and 1 ng in vitro transcribed firefly luciferase (*Luc*) RNA (prepared from a T7-driven *Luc* plasmid using the HiScribe™ T7 High Yield RNA Synthesis Kit) dissolved in RLT Plus Buffer (QIAGEN, Hilden, NRW, DE) were added to each sample prior to RNA isolation. Chloroform was added to the TRIzol containing samples, and the aqueous phase was used for RNA extractions using the RNeasy Mini Kit (74104, QIAGEN). Reverse transcription reactions were performed using the Maxima H Minus First Strand cDNA Synthesis Kit using random hexamers and oligo(dT)_18_ (K1652, Thermo Fisher Scientific) using a modified “RT-qPCR—First Strand cDNA Synthesis” protocol with optional 65 °C incubation, 0.5 µL Maxima RT per reaction, and a 30 min (50 °C) reaction time. Quantitative PCR was performed using GoTaq^®^ qPCR Master Mix (A6002, Promega, Madison, WI, USA) with 200 nM primers in 10 µL reactions, using primers to amplify portions of: HCoV-229E *ORF1* (229E-*ORF1*-F 5′-ATGCTCCGACGTTTGGACAT, 229E-*ORF1*-R 5′-GTACTACGACGACGAGCCTG), HCoV-229E *N* (229E-*N*-F 5′-GGCAAACGGGTGGATTTGTC, 229E-*N*-R 5′-CCCAGACGACACCTTCAACA), *GFP* (*GFP*-F 5′-CTTCTTCAAGTCCGCCATGC, *GFP*-R 5′-GGTCTTGTAGTTGCCGTCGT), and *Luc* (*Luc*-F 5′-GGCGAATTATGTGTCAGAGG, *Luc*-R 5′-TCCCAGTAAGCTATGTCTCC). Samples were run in triplicate in a CFX Connect Real-Time PCR Detection System (Bio-Rad) with the following two-step protocol: 3 min at 95 °C, 40 cycles of 10 s at 95 °C and 30 s at 60 °C. Data were analyzed using a ΔCt method; gene expression was normalized to “DMSO/Dark/Untreated” and expressed as fold change.

### 2.7. Pseudotyped Lentivirus Infection Assays

SARS-CoV-2 S-pseudotyped lentiviruses were generated as described in Section 2.1 using pLJM1-Luc2-BSD, pSPAX.2, and pcDNA3-Spike. pLJM1-Luc2-BSD was generated by subcloning Luc2 from the NheI and MfeI sites of pGL4.26 (Promega) into the NheI and EcoRI sites of pLJM1-BSD. pcDNA3-Spike encodes a human codon-optimized S matching bases 21,563 to 25384 from GenBank: NC_045512 (a kind gift from Dr. David Kelvin, Dalhousie University), with a D614G mutation, generated by PCR mutagenesis with primers: 5′-CTGTACCAGGgTGTGAACTGC and 5′-CACGGCCACCTGATTGCT using KOD Xtreme Hot Start DNA Polymerase (Sigma, 71975-M). Pseudotyped lentiviruses were treated with LED light at 49 J/cm^2^ after the addition of 4 µg/mL PhytoQuin or DMSO as described in Section 2.3, then added to HEK293A-ACE2/TMPRSS2 cells and incubated for 24 h at 37 °C. Cells were harvested in 1X Reporter Lysis Buffer (E397A, Promega, Madison, WI, USA) and lysed by freezing at −80 °C. Thawed lysates were added to an Falcon^®^ white/opaque 96-well plates (25382-208, VWR), mixed with Luciferase Assay System reagent (E1501, Promega) using automated injectors, and read on a FLUOstar Omega microplate reader (BMG Labtech).

### 2.8. Pseudotyped Lentivirus Purification and Western Blotting

SARS-CoV-2 S-pseudotyped lentiviruses were generated as indicated in Section 2.7 using pcDNA3-Spike, or pcDNA3-Spike-∆furin where the furin cleavage site was mutated (P681S, R682G, R683G, R685del) with primers: 5′-CTCCGTGGCAAGCCAGTCGATCATCGCCTAC, and 5′-GCGCCTCCACTCGAGTTCGTCTGGGTCTGGTA as described above. Following 0.45 µm filtration, the lentiviruses were purified through a 20% sucrose PBS cushion as described above in Section 2.5, then mixed with DMSO or 4 µg/mL PhytoQuin and incubated under LED light at 49 J/cm^2^ as indicated in Section 2.3. After treatment, the virions were harvested with 4X Laemmli Sample Buffer (250 mM Tris-Cl (pH 6.8), 8% sodium dodecyl sulfate, 40% glycerol, 50 mM dithiothreitol) to a final concentration of 1X and an incubation at 95 °C for 5 min. The samples and prestained protein standard (Broad Range (10–250 kDa); P7719, New England Biolabs, Ipswich, MA, USA) were loaded into 4–20% Mini-PROTEAN^®^ TGX Stain-Free™ Protein Gels (4568093, Bio-Rad, Hercules, CA, USA). Following SDS-PAGE, all proteins were transferred to polyvinylidene difluoride (PVDF) membranes using Trans-Blot Turbo LF PVDF Transfer Kit (1704275, Bio-Rad) and a Trans-Blot Turbo Transfer System (Bio-Rad). Membranes were blocked with 5% bovine serum albumin (BSA) in Tris-buffered saline/0.1% [vol/vol] Tween-20 (TBST) before probing with antibodies raised to the following targets: Anti-SARS-CoV-2 Spike RBD polyclonal antibody (E-AB-V1006, Elabscience, Houston, TX, USA) or anti-HIV1 p24 monoclonal antibody (ab9071, Abcam, Cambridge, UK). Membranes were washed with TBST, incubated with HRP-conjugated anti-rabbit IgG (7074, Cell Signaling, Danvers, MA, USA) or anti-mouse IgG (7076, Cell Signaling) secondary antibodies diluted in 5% BSA/TBST, and washed again with TBST prior to detection with Clarity Western ECL Substrate (1705061, Bio-Rad). All blots were imaged on a Bio-Rad ChemiDoc-Touch system.

### 2.9. Data Management and Statistics

All data management was performed with Microsoft^®^ Excel^®^ for Microsoft 365. Graphing and statistical calculations were performed using GraphPad Prism v9.2.0. The final figures were prepared using Affinity Designer v1.10.1.1142.

## 3. Results

### 3.1. Measurement of Phototoxic Threshold of PhytoQuin in Huh7.5 Cells and BHK-21 Cells

Photodynamic inactivation (PDI) employs a photosensitizer (PS), light, and oxygen to create a local burst of reactive oxygen species (ROS) that inactivate microbes. For our experiments to investigate the PDI of human coronaviruses, we employed a PS, PhytoQuin, which is an extract from *Polygonum cuspidatum* (common name: Japanese Knotweed) prepared by PhotoDynamic, Inc. We began by evaluating cell viability with and without light exposure following treatment with PhytoQuin or the solvent, dimethyl sulfoxide (DMSO). We chose Huh7.5 (human hepatocellular carcinoma) cells and baby hamster kidney-21 (BHK-21) cells for these analyses because they are susceptible to infection by HCoV-229E and HCoV-OC43, respectively.

**Figure 1:**
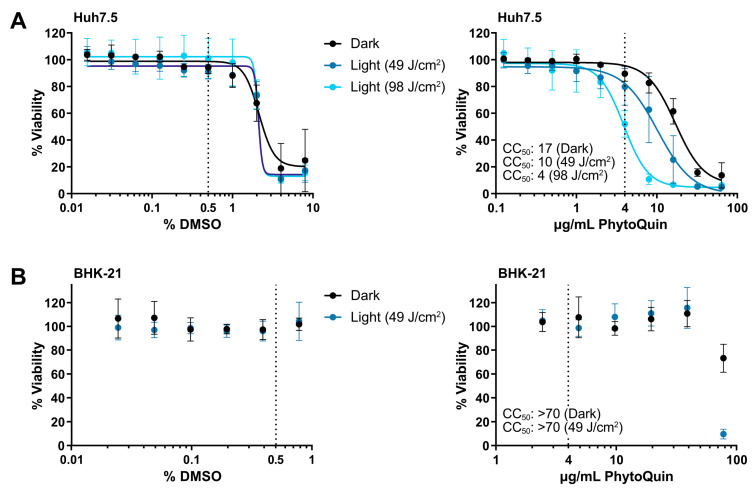
Cell viability following DMSO and PhytoQuin treatment of Huh7.5 and BHK-21 cells. Huh7.5 cells (**A**) or BHK-21 cells (**B**) in low-serum infection media were exposed to increasing concentrations of DMSO (solvent, left panels) or PhytoQuin (right panels) with (49 J/cm^2^, blue points; 98 J/cm^2^, light blue points) or without (black points) LED light treatment. Cell viability was assayed at 48 h post-treatment using alamarBlue cell viability reagent. The vertical dotted lines indicate the highest concentrations of DMSO and PhytoQuin used in subsequent experiments. Data are plotted as averages ± SEM from three independent experiments. The 50% cytotoxic concentration (CC_50_) values were calculated using Prism v9.2.0. Abbreviations: DMSO, dimethyl sulfoxide; LED, light-emitting diode; SEM, standard error of the mean.

To establish the phototoxic threshold concentrations of PhytoQuin, Huh7.5 cells were treated with PhytoQuin or vehicle control at room temperature with exposure to LED light (49 J/cm^2^ or 98 J/cm^2^) or protected from light followed by culture at 37 °C for 48 h. DMSO concentrations above 2% were toxic to Huh7.5 cells where no additional toxicity was observed with either light treatment (Figure 1A). The 50% cytotoxic concentration (CC_50_) of PhytoQuin in Huh7.5 cells was 10 μg/mL at 49 J/cm^2^ of photoactivation and 4 μg/mL after 98 J/cm^2^ of photoactivation (Figure 1A). PhytoQuin was less toxic in the absence of photoactivation (“Dark”), with a CC_50_ of 17 μg/mL. BHK-21 cells behaved similarly with DMSO treatment, with no cytotoxicity up to the highest concentration tested (0.8%; Figure 1B). While only 49 J/cm^2^ of light exposure was tested using BHK-21 cells, cytotoxicity was only observed with PhytoQuin concentrations of 78 µg/mL, suggesting that the BHK-21 cells are more resistant to transient ROS-mediated damage than Huh7.5 cells (Figure 1B). Altogether, these experiments demonstrate the low cytotoxicity of PhytoQuin in these human cell lines. A concentration of 4 µg/mL PhytoQuin (equivalent to 0.5% DMSO, indicated by the vertical dashed lines in Figure 1) was selected as the maximal dose used in all subsequent infection experiments, which falls well below the CC_50_ value for both cell lines.

### 3.2. PhytoQuin Exhibits Light-Dependent Antiviral Activity against HCoV-229E and HCoV-OC43

**Figure 2:**
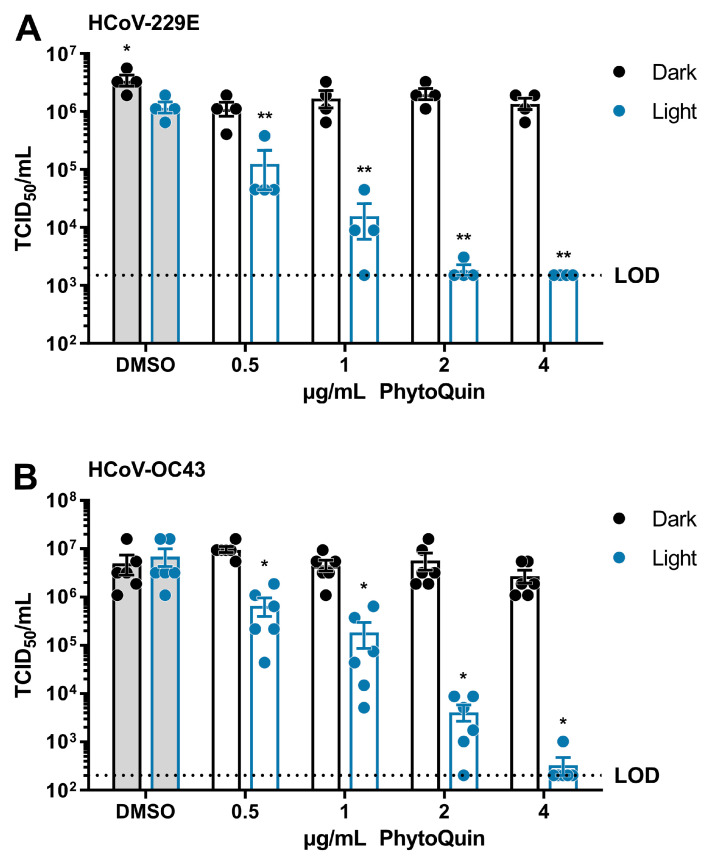
PhytoQuin potently inactivates human coronaviruses 229E and OC43. (**A**) HCoV-229E was combined with 0.5% DMSO or PhytoQuin (0.5, 1, 2, or 4 µg/mL) prior to exposure to LED light in black tubes (“Dark”, black dots/lines) or clear tubes (“Light”, blue dots/lines) at 49 J/cm^2^. The treated inocula were serially diluted and used to infect Huh7.5 cells and titered using TCID_50_ assays. (**B**) HCoV-OC43 was combined with 0.5% DMSO or PhytoQuin (0.5, 1, 2, or 4 µg/mL) prior to exposure to light as indicated for panel A. The treated inocula were serially diluted and used to infect BHK-21 cells and titered using TCID_50_ assays. The resulting TCID_50_/mL values are plotted showing the individual data points with each bar graph (DMSO, grey; PhytoQuin, white) representing the average ± SEM from four or six independent experiments. The LOD for the TCID50 assays is indicated by the horizontal dotted line. * *p* = 0.01–0.05, ** *p* = 0.001–0.01, using unpaired *t*-tests comparing to DMSO/+Light. Abbreviations: DMSO, dimethyl sulfoxide; LED, light-emitting diode; LOD, limit of detection; SEM, standard error of the mean; TCID_50_, median tissue culture infectious dose.

Having established the baseline phototoxic threshold of PhytoQuin in Huh7.5 and BHK-21 cells, we next measured the antiviral activity of PhytoQuin against HCoV-229E and HCoV-OC43. HCoV-229E inocula were mixed with the indicated concentrations of DMSO or PhytoQuin and exposed to light at 49 J/cm^2^, or kept in the dark, before being serially diluted for infecting Huh7.5 cells. The remaining viral infectivity after each treatment was determined using median tissue culture infectious dose (TCID_50_) assays. By exposing the HCoV-229E viral inoculum to DMSO and light, we observed that HCoV-229E is mildly photosensitive to 49 J/cm^2^ LED exposure, displaying <3-fold decrease in viral titers compared to DMSO/Dark treated virus (Figure 2A). PhytoQuin incubated with HCoV-229E in the dark had little effect (<3-fold decrease relative to DMSO/Dark) across the full range of concentrations tested (Figure 2A). By contrast, a clear, dose-dependent reduction in viral titers was observed between 0.5 μg/mL and 4 μg/mL doses of photoactivated PhytoQuin; with all samples at the 4 μg/mL dose reducing viral titers to the limit of detection (LOD, 1500 TCID_50_/mL) of the assay (Figure 2A). These data demonstrate that HCoV-229E is susceptible to PDI, with ~1000-fold reductions in viral titers after a brief light treatment.

To determine whether the antiviral effects of PhytoQuin PDI were specific for the alphacoronavirus HCoV-229E or generalizable across diverse HCoVs, we next tested its activity on the distantly related betacoronavirus, HCoV-OC43. The viral inocula were exposed to sub-cytotoxic doses of 0.5–4 µg/mL PhytoQuin, either with or without 49 J/cm^2^ LED light exposure, and then used to infect BHK-21 cells for TCID_50_ assays. Compared to HCoV-229E, similar amounts of HCoV-OC43 were inactivated by PhytoQuin in a dose-dependent and light-dependent manner (Figure 2B). PhytoQuin (4 µg/mL) and light treatment led to an approximately 4 log_10_ reduction in viral titer relative to DMSO-treated HCoV-OC43. Together, these data show that various HCoVs are highly susceptible to PhytoQuin PDI.

### 3.3. PhytoQuin Treatment Does Not Cause Visible Damage to HCoV Particles

Photoactivation of PhytoQuin generates ROS that can damage nearby macromolecules, which is thought to be the primary antimicrobial mechanism of PhytoQuin PDI. HCoVs have a lipid envelope studded with viral glycoproteins required for key steps in cellular entry. We previously observed that PhytoQuin PDI did not damage herpesvirus DNA genomes [20] that are well protected by a strong icosahedral capsid [23]; we reasoned that the HCoV RNA genome, which is only lightly protected by viral nucleocapsid (N) proteins, may be more susceptible to PDI. Specifically, we hypothesized that PhytoQuin PDI may damage HCoV lipid envelopes and render the RNA genome more susceptible to damage or degradation.

**Figure 3:**
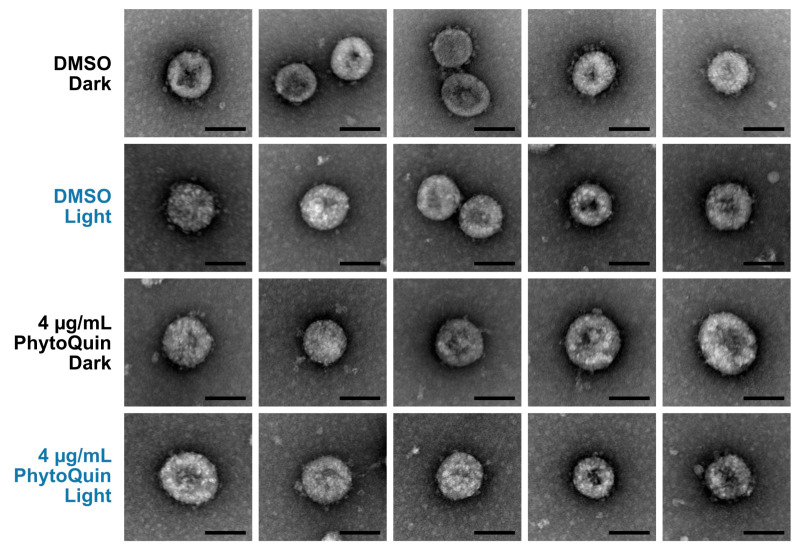
PhytoQuin treatment does not cause overt damage to HCoV-OC43 particles. HCoV-OC43 particles treated with 0.5% DMSO (+/−LED light) or 4 µg/mL PhytoQuin (+/−LED light) were fixed, negative stained with uranyl acetate, and imaged using a transmission electron microscope. Five to seven viral particles are shown per condition. Scale bar = 100 nm. Abbreviations: DMSO, dimethyl sulfoxide; LED, light-emitting diode.

We first examined PhytoQuin-treated HCoV virions by transmission electron microscopy (TEM) to determine whether treatment caused any gross alterations in virion morphology that could represent virion damage. HCoV-OC43 virions were treated with 4 μg/mL of PhytoQuin in the presence of light at 49 J/cm^2^, which we previously demonstrated was sufficient to inhibit viral replication (Figure 2B). In parallel, virus preparations were treated with PhytoQuin in the dark or treated with DMSO vehicle control. Following treatment, samples were fixed, negative-stained, and mounted on grids for TEM. We observed that viral particles treated with DMSO or PhytoQuin, irrespective of exposure to light, were consistent in size and shape across all conditions tested (Figure 3). Using our staining procedure, glycoprotein spikes were visible on the surface of some but not all virions, with these being most evident in the DMSO-treated samples (Figure 3). However, our limited sample size made it difficult to quantify these subtle morphological changes following PDI, and no causal relationship could be established between these morphological changes and loss of infectivity. Based on these observations, we concluded that even though PhytoQuin PDI caused a ~4-log drop in infectivity, it did not cause any gross structural damage to CoV virions.

### 3.4. PhytoQuin PDI of HCoV Virions Damages the Viral Lipid Envelope

**Figure 4:**
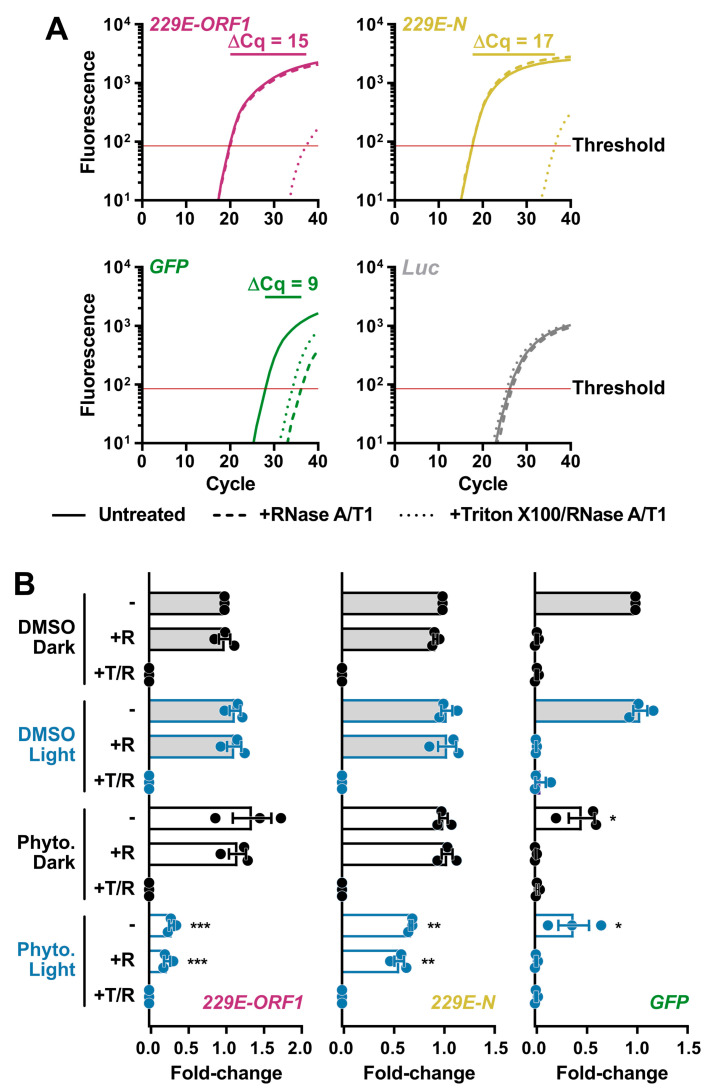
Light-activated PhytoQuin damages HCoV-229E lipid envelopes and renders vRNA susceptible to degradation by RNases. (**A**) Development of an RNase protection assay to quantitate vRNA levels in HCoV-229E inocula. Virions in serum-free medium were combined with in vitro transcribed *GFP* RNA and left untreated (—), treated with RNase A/T1 (---), or lysed with Triton X-100 prior to RNase A/T1 treatment (···). The remaining RNA was combined with *Luc* RNA and was extracted, reverse transcribed, and quantitated using qPCR to measure the viral sequences, *ORF1* (red) and *N* (yellow), and the control *GFP* (green) or *Luc* (grey) cDNAs. The averaged amplification curves from one representative experiment performed in triplicate from three independent replicates are shown with the difference in Cq values between untreated and Triton + RNase treatment indicated above the curves. (**B**) HCoV-229E virions were combined with 0.5% DMSO or 4 µg/mL PhytoQuin prior to exposure to LED light in black tubes (Dark, black dots/lines) or clear tubes (Light, blue dots/lines) at 49 J/cm^2^. The viral particles were then left untreated (−), treated with RNase A/T1 (+R), or treated with both Triton X-100 and RNase A/T1 (+T/R) prior to RNA extraction, reverse transcription, and qPCR analysis. The fold-changes in cDNA levels were plotted relative to the DMSO/Dark-treated samples showing the individual data points from three independent experiments with each bar graph (DMSO, grey; PhytoQuin, white) representing averages ± SEM. * *p* = 0.01–0.05, ** *p* = 0.001–0.01, *** *p* = 0.001–0.0001 using unpaired *t*-tests compared to the respective DMSO control. Abbreviations: cDNA, complementary DNA; Cq, quantification cycle; DMSO, dimethyl sulfoxide; *GFP*, green fluorescent protein; LED, light-emitting diode; *Luc*, luciferase; N, nucleocapsid; ORF1, open-reading frame; Phyto., PhytoQuin; SEM, standard error of the mean; T, Triton X-100; R/RNase, ribonuclease; vRNA, viral RNA.

Even though PhytoQuin PDI did not cause gross morphological changes in HCoV particles, we reasoned that PDI could nevertheless breach the lipid envelope and render the genome susceptible to degradation by ribonucleases (RNases). We developed an RNase protection assay for measuring vRNA levels in virions to monitor for PDI-mediated disruption of the CoV lipid envelope. To establish the parameters for the assay, HCoV-229E virions in infected cell supernatants were incubated in the presence or absence of RNase at 37 °C for 20 min or incubated with RNase in the presence of Triton X-100, a non-ionic detergent that disrupts lipid bilayers like the viral envelope. After a 20 min incubation, RNase activity was inhibited using TRIzol, and viral RNA was isolated and reverse transcribed. As a positive control, a synthetic *GFP* RNA was included prior to the RNase treatments as an unprotected RNA species susceptible to RNase activity. A synthetic *Luc* RNA was added post-TRIzol extraction to confirm RNase inhibition by monitoring for undesired RNA degradation following TRIzol disassociation of the virions/viral ribonucleoprotein (vRNP) complexes. qPCR was performed using oligonucleotide primers that amplify portions of the HCoV-229E *N*, HCoV-229E *ORF1*, *GFP*, or *Luc* genes. Our pilot experiments validated that vRNA was protected from exogenous RNase activity by the viral lipid envelope and was only susceptible to RNase activity after the viral lipid envelope was disrupted (Figure 4A); as indicated by the increase in Cq values after Triton X-100 addition. *GFP* RNA was immediately susceptible to degradation by RNase whereas little effect was observed on *Luc* RNA which was added after RNase inhibition (Figure 4A). The higher abundance (earlier amplification) of HCoV-229E *N* relative to *ORF1* may be due to the presence of viral subgenomic RNA (sgRNA) in exosomes, as this has also been observed in SARS-CoV-2 qPCR experiments [24].

This assay was subsequently used to determine whether damage induced by PhytoQuin PDI breaches the HCoV lipid envelope. HCoV-229E stocks were incubated with 4 μg/mL of PhytoQuin in the presence of LED light at 49 J/cm^2^, which we previously demonstrated was sufficient to inactivate HCoV-229E (Figure 2A). In parallel, virion preparations were treated with PhytoQuin in the dark or treated with DMSO vehicle control with and without light exposure. All samples were then treated with RNase +/− Triton X-100 for 20 min at 37 °C or left untreated. This analysis revealed that PhytoQuin treatment alone, in the absence of light treatment, did not render the viral genome more susceptible to RNase digestion (Figure 4B). However, when light treatment was coupled with PhytoQuin treatment, significant reductions in *N* and *ORF1* RNA copies were observed (Figure 4B). Strikingly, the loss of *N* or *ORF1* RNA occurred irrespective of the addition of exogenous RNase (Figure 4B), suggesting that either there was a low level of RNase contamination in the PhytoQuin extract or that PhytoQuin, or the ROS generated from it, can directly damage the vRNA. As we observed an equivalent decrease in *GFP* levels following PhytoQuin addition with or without photoactivation (Figure 4B), this suggests that some component of the extract can damage RNA directly in a light-independent manner. Overall, it is clear from these data that PhytoQuin PDI damages enveloped particles in a manner that renders HCoV RNA susceptible to degradation.

### 3.5. PhytoQuin PDI Prevents Infection of ACE2/TMPRSS2-Expressing Cells by SARS-CoV-2 S-Pseudotyped Lentiviruses by Altering S Glycoproteins

**Figure 5:**
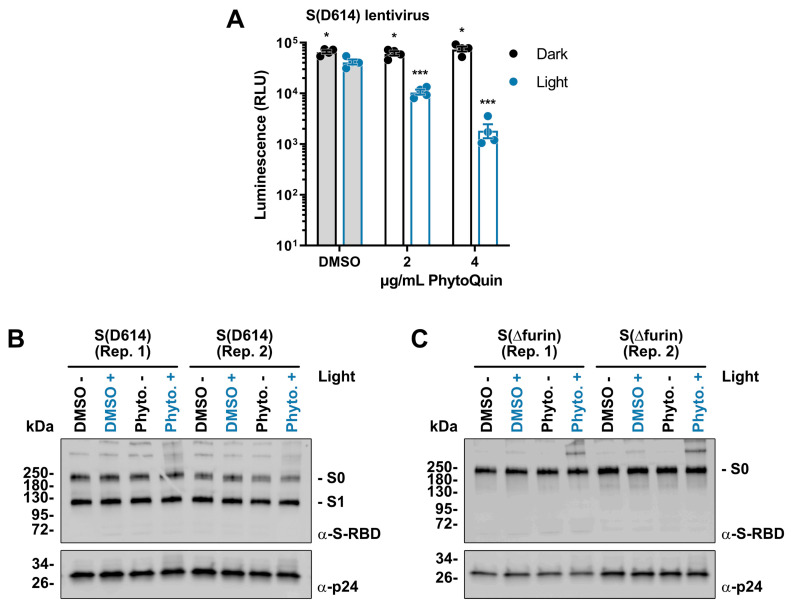
Light-activated PhytoQuin inhibits SARS-CoV-2 S-pseudotyped lentivirus entry and alters S protein electrophoretic mobility. (**A**) SARS-CoV-2 S (D614)-pseudotyped lentiviruses were combined with 0.5% DMSO or PhytoQuin (2 or 4 µg/mL) prior to exposure to LED light in black tubes (Dark, black dots/lines) or clear tubes (Light, blue dots/lines) at 49 J/cm^2^. The treated lentiviruses were then used to transduce ACE2/TMPRSS2-expressing 293A cells, and luciferase transgene expression was measured 48 h post-transduction. The resulting relative luminescence units (RLU) are plotted showing the individual data points with each bar graph (DMSO, grey; PhytoQuin, white) representing the average ± SEM from four independent experiments. * *p* = 0.01–0.05, *** *p* = 0.0001–0.001 using unpaired *t*-tests comparing to DMSO/+Light. Two independent batches of SARS-CoV-2 S (D614)-pseudotyped lentiviruses (**B**) or SARS-CoV-2 S (Δfurin)-pseudotyped lentiviruses (**C**) were purified via ultracentrifugation and then combined with 0.5% DMSO or 4 µg/mL PhytoQuin prior to exposure to light in black tubes (Light: −/black font) or clear tubes (Light: +/blue font) at 49 J/cm^2^. After treatment, lentiviral particles were solubilized in 2X Laemmli buffer and subjected to SDS-PAGE followed by immunoblotting to visualize SARS-CoV-2 S protein (α-S-RBD) and HIV-1 p24 (α-p24). Abbreviations: DMSO, dimethyl sulfoxide; kDa, kilodalton; LED, light-emitting diode; Phyto., PhytoQuin; RBD, receptor binding domain; Rep, replicate; SEM, standard error of the mean; S, spike.

Having established that PhytoQuin PDI damages the viral lipid envelope, we next sought to determine whether viral glycoproteins could also be affected by PDI. As we lacked antibodies to visualize HCoV-229E or OC43 Spike (S), we created lentiviruses pseudotyped with SARS-CoV-2 S for which Western blotting reagents were readily available. These lentiviruses also carried a firefly luciferase gene to measure infection. Lentiviruses were incubated with 2–4 μg/mL of PhytoQuin (or equivalent DMSO) in the presence or absence of LED light at 49 J/cm^2^. Following treatment, the lentiviruses were used to infect human HEK 293A cells co-expressing angiotensin-converting enzyme 2 (ACE2) and transmembrane serine protease 2 (TMPRSS2), which act as a key receptor and accessory factor, respectively, for SARS-CoV-2 S-mediated entry [25,26,27]. Light treatment in the presence of DMSO led to <1.5-fold decrease in luciferase expression, demonstrating that lentiviruses also have a small degree of light sensitivity (Figure 5A), similar to HCoVs (Figure 2A and Figure 3B). Importantly, we observed that PhytoQuin PDI strongly inhibited luciferase gene expression in a light-dependent and dose-dependent manner (Figure 5A). This suggests that either PDI interferes with S interactions with the cell surface or that the lentiviral lipid envelope is disrupted in such a way as to prevent proper delivery of the luciferase reporter gene to the cell nucleus. Regardless, these data provide further evidence for the antiviral potency of PhytoQuin PDI and support our use of this model to study the effects of PhytoQuin on viral glycoproteins.

Since viral glycoproteins stud the surface of enveloped viruses like HCoVs, they are particularly susceptible to damage initiated from the extracellular environment. This would include any oxidative damage to viral glycoproteins caused by PhytoQuin PDI. We used our pseudotyped lentiviruses to investigate any changes in protein integrity potentially caused by PhytoQuin using Western blotting. Using wild-type, S (D614)-pseudotyped lentiviruses, we demonstrated that treating samples with PhytoQuin and light caused alterations in S protein electrophoretic mobility, with the emergence of slow-migrating forms of S with abnormally high predicted molecular weights (Figure 5B). SARS-CoV-2 S, like other CoV S proteins, is processed by host proteases into distinct molecules, namely, S1 and S2, with S0 being the full-length precursor [25]. We next generated additional lentiviruses pseudotyped with a mutant S that lacks the furin cleavage site (S(Δfurin)), to better visualize differences in S by concentrating the detected protein into a single species, the precursor S0. Using the same experimental set-up, we again observed that photoactivated PhytoQuin caused the emergence of S protein isoforms with reduced electrophoretic mobility (Figure 5C). These observations, together with our data showing PhytoQuin-mediated effects on HCoV lipid envelopes and vRNA, demonstrate that PhytoQuin PDI can damage multiple components of HCoV particles.

### 3.6. Emodin Is a Significant Contributor to the Antiviral Activity of PhytoQuin

**Figure 6:**
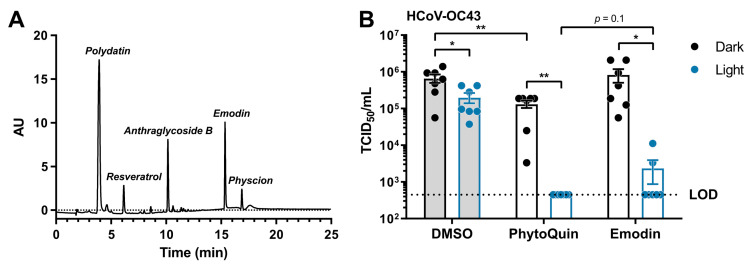
Light-activated emodin is sufficient to inactivate HCoV-OC43. (**A**) HPLC trace identifying the constituents of the current lot of PhytoQuin. AU, absorbance units. (**B**) HCoV-OC43 was combined with 0.5% DMSO, 4 µg/mL PhytoQuin, or 117 ng/mL emodin prior to exposure to LED light in black tubes (“Dark”, black dots/lines) or clear tubes (“Light”, blue dots/lines) at 49 J/cm^2^. The treated inocula were serially diluted and used to infect cells in 96-well plates to assay for infectivity using TCID_50_ assays. The resulting TCID_50_/mL values are plotted showing the individual data points with each bar graph (DMSO, grey; PhytoQuin, white) representing the average ± SEM from seven independent experiments. The LOD for the TCID_50_ assays is indicated by the horizontal dotted line. * *p* = 0.01–0.05, ** *p* = 0.001–0.01 using unpaired *t*-tests with the indicated comparisons. Abbreviations: AU, absorbance units; DMSO, dimethyl sulfoxide; LED, light-emitting diode; LOD, limit of detection; SEM, standard error of the mean; TCID_50_, median tissue culture infectious dose.

PhytoQuin is a complex botanical extract with multiple constituents that could, individually or synergistically, contribute to its antiviral activity. HPLC analysis of the current lot of PhytoQuin identified five principal constituents, in order of abundance as follows: polydatin, anthraglycoside B, emodin, resveratrol, and physcion (Figure 6A). Emodin is an anthraquinone and PS that when photoactivated can generate ROS [28] and is abundant in *Polygonum cuspidatum* extracts. After observing that PhytoQuin PDI damaged HCoV lipid envelopes and considering that emodin affects membrane fluidity and integrity [29], we investigated whether emodin alone could recapitulate the PhytoQuin PDI antiviral effect against CoVs.

From our HPLC analysis, we observed that the amount of emodin, as compared to a commercial emodin standard, equaled 29.3 mg per gram of extract (~3% *w*/*w*). To investigate whether emodin is a key PS required for PhytoQuin PDI against HCoVs, we treated HCoV-OC43 inoculum with a dose of photoactivated emodin (117 ng/mL) that matched its concentration in a 4 µg/mL PhytoQuin extract. In these experiments we observed a three-fold inhibition of HCoV-OC43 infectivity due to LED light treatment alone (Figure 6B), similar to the inhibition observed for HCoV-229E when exposed to LED light (Figure 2A). While emodin treatment in the absence of light had no impact on HCoV-OC43 titers, PhytoQuin treatment without photoactivation did have a small impact on HCoV-OC43 infectivity (Figure 6B). Photoactivated PhytoQuin resulted in 99.8% reduction in HCoV-OC43 titre compared to DMSO-treated virus exposed to light (Figure 6B). When treated with LED light, emodin caused a similar 98.5% reduction in HCoV-OC43 titer compared to the DMSO + Light control, where the inhibitory effect of emodin was indistinguishable from that of PhytoQuin (Figure 6B). Therefore, these data indicate that emodin is a significant contributor to the antiviral activity of PhytoQuin against human coronaviruses.

## 4. Discussion

The COVID-19 pandemic has provided new motivation to discover and develop antivirals against coronaviruses. We demonstrated that PhytoQuin, a photosensitizing botanical extract, inactivated distantly related human coronaviruses HCoV-229E and HCoV-OC43, as well as pseudotyped lentiviruses bearing SARS-CoV-2 S protein. PhytoQuin acts in a light-dependent manner to damage nearby macromolecules, which is consistent with a ROS-mediated mechanism of action as observed for other photosensitizers. Our study provides strong support for the addition of PhytoQuin and the PhytoQuin constituent emodin to the growing list of PSs with antiviral activity against CoVs [30,31,32,33,34,35,36,37].

We observed that while PhytoQuin PDI did not cause gross structural changes in viral particles (Figure 3), it did disrupt the viral lipid envelope to expose the viral genome to ribonucleases (Figure 4) and altered the electrophoretic mobility of S proteins (Figure 5). These changes strongly correlated with diminished infectivity of CoVs and S-pseudotyped lentiviruses following PhytoQuin PDI (Figure 2 and Figure 5A). Unlike within the cell where quenching by antioxidants can occur, free radicals generated in the extracellular milieu have unfettered access to damage viral structural proteins and inactivate viruses. Certain surface-exposed amino acid side chains are sensitive to oxidative damage, which can result in peptide bond cleavage, amino acid modifications, and intra- and inter-protein crosslinking [38]. Our observation of reduced electrophoretic mobility of SARS-CoV-2 S from purified lentiviruses following exposure to photoactivated PhytoQuin is suggestive of aggregation or cross-linking of these viral glycoproteins (Figure 5B,C). CoV S proteins contain numerous cysteine residues in their ectodomains, where the majority form disulfide bonds to support protein structure and function [39,40,41,42,43,44]. Cysteines of S proteins may be oxidized via photoactivation to form inter-protein linkages similar to heterodimeric protein crosslinking observed in vitro with the PS rose bengal [45]. Furthermore, multi-step oxidation reactions particularly of Tyr, Trp, Met, and His residues can generate reactive peroxyl or carbonyl derivatives that react with nucleophilic protein side chains to crosslink proteins [46]. Spurious crosslinking of proximal S proteins may reduce protein flexibility and interfere with conformational changes required for receptor interactions and membrane fusion [47]. The appearance of new S protein species with reduced electrophoretic mobility under reducing conditions suggests that changes in S due to PhytoQuin treatment are likely linked to irreversible protein cross-linking. The precise covalent modifications of S following exposure to photoactivated PhytoQuin remain unknown, but ongoing experiments will investigate whether PDI increases protein carbonylation, a hallmark of protein oxidative damage, using 2,4-dinitrophenylhydrazine-based assays.

Multiple mechanisms exist to mitigate ROS-induced changes in membrane integrity [48], which could alleviate any negative impacts of brief PhytoQuin treatments on living cells. Conversely, viral particles cannot repair oxidative damage. The most striking impact of PhytoQuin treatment on HCoV particles was the loss of vRNA after light treatment (Figure 4B), which is likely a direct result of ROS damage to the viral lipid envelope, which would expose the vRNP complexes to damaging RNases. While oxidation is known to directly damage nucleic acids [16], our current experiments did not measure whether the ROS produced during PhytoQuin-mediated PDI result in direct damage to nucleic acids. However, disruptive effects on cellular and liposomal membranes due to light-activated PSs have been previously documented [49,50,51,52]. In our previous work, we did not observe any effect of PhytoQuin on herpesvirus DNA when analyzing viral DNA copy number by qPCR following sequential PhytoQuin and deoxyribonuclease treatment [20]. This may be due to the presence of a more robust icosahedral capsid surrounding the DNA genome of herpesviruses compared to the more flexible, N-associated vRNPs of HCoVs. However, PhytoQuin likely also damages herpesvirus envelopes as earlier work demonstrated the ability of anthraquinones to disrupt HSV-1 virions [53]. Evidence of physical damage to viral particles due to PhytoQuin treatment was further supported by the appearance of high molecular weight forms of SARS-CoV-2 S on purified, pseudotyped lentiviruses following light exposure (Figure 5). Taken together, our data provide strong evidence for a light-dependent mechanism of action that involves damage to the virion exterior, to lipids and proteins alike, resulting in the inhibition of viral infection.

While seemingly complex, PhytoQuin is primarily composed of emodin and resveratrol and their naturally occurring derivatives (Figure 6A). Polydatin is the precursor of resveratrol, and anthraglycoside B or physcion are glucopyranoside or methyl ether derivatives of emodin, respectively. Emodin and resveratrol are known antiviral polyphenolic compounds which inhibit the replication of a number of respiratory viruses, including influenza A virus [54,55], rhinovirus [56], and respiratory syncytial virus [57,58,59], as well as several HCoVs including MERS-CoV, HCoV-229E and SARS-CoV-2 [60,61,62,63,64]. Despite this, there is no clear consensus on antiviral mechanism of action of resveratrol or emodin. Resveratrol and aloe-emodin, an isomer of emodin, were recently shown to be antimicrobial PSs [65,66,67]. These studies used blue LED photoactivation with comparable fluence to the white light LED source used herein; however, the concentrations for antimicrobial activity of photoactivated emodin (≥27 µg/mL) or resveratrol (2 mg/mL) were significantly higher than those in 4 µg/mL of PhytoQuin. Our current study provides strong evidence that emodin is a photoactivatable antiviral that can be added to a growing list of antiviral PSs [68]. The near-complete inactivation of HCoV-OC43 by emodin (Figure 6B) suggests that, while a predominant factor, other constituents of the extract such as resveratrol may have minor contributions to PhytoQuin PDI. Interestingly, our previous work demonstrated similar antiviral effects against herpesviruses and adenoviruses by PhytoQuin [20], but at doses four to ten-fold lower than those used herein for HCoVs. The HPLC trace of the original lot of PhytoQuin demonstrated a significantly higher concentration of emodin compared to the lot employed for our current CoV experiments. It is not yet known if emodin is also the primary bioactive component of PhytoQuin for neutralizing herpesviruses, adenoviruses, rhabdoviruses, or bacteria. Future experiments are needed to evaluate this possibility. It would be interesting to screen various lots or differentially extracted PhytoQuin extracts to help determine if emodin content positively correlates with antiviral or antibacterial activity.

Many of the above-mentioned studies investigated the pleiotropic antiviral activities of emodin, resveratrol, and other polyphenolics following addition to infected cells. In this study, we show that the PhytoQuin extract can directly inactivate viruses in the extracellular environment through oxidative damage catalyzed by light treatment. Since the PhytoQuin or emodin were not removed from the treated inocula prior to infection, we cannot exclude the possibility that these compounds may have some impact on the cells as well. However, our characterization of viral particles after treatment with photoactivated PhytoQuin via qPCR and Western blotting clearly shows deleterious physical changes to the virions. PhytoQuin or emodin treatment in the absence of light exposure showed little to no effect on HCoV infectivity or integrity, nor was PhytoQuin cytotoxic in the presence or absence of light at a final concentration of 4 µg/mL. Therefore, it is possible that the working concentrations of emodin or resveratrol present in 4 µg/mL of PhytoQuin may be low enough to avoid the cell-intrinsic effects previously attributed to these two constituents. Exploiting their photoreactive properties can help to lower the effective working antimicrobial concentrations of emodin and resveratrol. Controlling light exposure will be an important consideration in future studies into the antiviral effects of polyphenols and anthraquinones.

Our observation that PhytoQuin-mediated PDI is effective at neutralizing bacteria [18], herpesviruses, adenoviruses, rhabdoviruses [20], and now HCoVs and lentiviruses highlights the broad photodynamic activity of this botanical extract. Indeed, by demonstrating broad antiviral activity against distantly related HCoVs, 229E and OC43, as well as the formation of higher molecular weight isoforms of S that correlate with the disruption of S-pseudovirus interactions with ACE2 receptors, our work suggests that PhytoQuin PDI may be effective against future emerging CoVs with similar physical properties. In the meantime, ongoing work will focus on further elucidation of the mechanism of action of PhytoQuin PDI against known CoVs, while optimizing the formulation to improve antiviral specificity, efficacy, and duration of effect.

## Data Availability

Not applicable.
